# Estimation of Genetic Parameters for Pork Quality, Novel Carcass, Primal-Cut and Growth Traits in Duroc Pigs

**DOI:** 10.3390/ani10050779

**Published:** 2020-04-30

**Authors:** Hannah E. Willson, Hinayah Rojas de Oliveira, Allan P. Schinckel, Daniela Grossi, Luiz F. Brito

**Affiliations:** 1 Department of Animal Sciences, Purdue University, West Lafayette, IN 47907, USA; hwillso@purdue.edu (H.E.W.); holivier@uoguelph.ca (H.R.d.O.); aschinck@purdue.edu (A.P.S.); 2 Fast Genetics, Saskatoon, SK S7K 2K6, Canada; dgrossi@fastgenetics.com

**Keywords:** belly trait, genetic correlation, heritability, meat quality, pork color, swine

## Abstract

**Simple Summary:**

There is a growing interest in worldwide swine breeding programs to genetically select for pork quality and primal cuts in addition to the traditional growth and carcass leanness traits. Accurate population genetic parameters are needed to estimate correlated responses to selection and incorporate novel traits in the selection objective. Therefore, we estimated heritabilities and genetic correlations for 39 pork quality, growth and carcass traits in Duroc pigs. In general, moderate and favorable genetic correlations were observed between pork quality (e.g., loin color and marbling scores) and carcass traits. Additionally, moderate to low correlations were found among pork quality, growth and carcass traits. Our findings suggest that pig breeders can successfully incorporate pork quality and novel carcass traits in the selection objectives without undesirable impacts upon growth rate and carcass leanness.

**Abstract:**

More recently, swine breeding programs have aimed to include pork quality and novel carcass (e.g., specific primal cuts such as the Boston butt or belly that are not commonly used in selection indexes) and belly traits together with growth, feed efficiency and carcass leanness in the selection indexes of terminal-sire lines, in order to efficiently produce pork with improved quality at a low cost to consumers. In this context, the success of genetic selection for such traits relies on accurate estimates of heritabilities and genetic correlations between traits. The objective of this study was to estimate genetic parameters for 39 traits in Duroc pigs (three growth, eight conventional carcass (commonly measured production traits; e.g., backfat depth), 10 pork quality and 18 novel carcass traits). Phenotypic measurements were collected on 2583 purebred Duroc gilts, and the variance components were estimated using both univariate and bivariate models and REML procedures. Moderate to high heritability estimates were found for most traits, while genetic correlations tended to be low to moderate overall. Moderate to high genetic correlations were found between growth, primal-cuts and novel carcass traits, while low to moderate correlations were found between pork quality and growth and carcass traits. Some genetic antagonisms were observed, but they are of low to moderate magnitude. This indicates that genetic progress can be achieved for all traits when using an adequate selection index.

## 1. Introduction

The North American swine industry produces over 146 million pigs per year, which accounts for over 15 million tons of pork products [1]. Pork production in the United States generated over 21 billion dollars in gross income from meat sales in 2018 [2], which is expected to increase due to a greater demand for pork [3,4]. In order to meet this growing demand, worldwide swine breeding programs have focused upon increasing the lean growth rate and feed efficiency of pigs by selecting for rapid growth and reduced backfat depth [5,6,7].

Currently, consumers desire high-quality meat products, which are perceived as more tender, juicier and possessing of desirable flavor [8,9]. Pork quality in this context can be defined as consumers’ sensory acceptance of pork products, which have a stable pH, pinkish-red color and moderate marbling (i.e., intra-muscular fat) content [8,10,11]. Therefore, it is becoming important to include pork quality with growth and carcass leanness traits in the selection objectives in order to attend to consumers’ growing demands for higher quality pork [8,12,13]. Additionally, as the value of primal cuts (e.g., picnic shoulder, Boston butt, loin, ham and belly) increases, producers need to consider additional carcass traits when making selection decisions to increase carcass cut-out value. 

Pork quality traits have been previously estimated to have low to moderate heritabilities, while carcass traits have moderate to high heritabilities [13,14,15,16]. However, there are few [13,14,16] to no previously estimated genetic parameters for primal and subprimal carcass cuts with pork quality and growth traits measured in the same population. For instance, traits such as the belly flop test, belly width and belly length have not been previously genetically evaluated, and the difference between trimmed and untrimmed primal cuts has only been evaluated in a crossbred population [13]. As crossbred animals are used primarily for meat products, it is important to understand the genetics of their pork quality. However, it is also valuable to estimate meat quality and carcass traits in the terminal sire line as, within swine breeding, the sire line’s most valuable contribution is their meat quality and carcass attributes. Duroc is the most common terminal sire line in North America, likely due to their high pork quality (darker meat with more marbling) and fast rate of growth [17,18,19]. Favorable genetic correlations between these traits may exist and could enable simultaneous selection for improved pork quality and primal-cut yield. However, there could also be unfavorable correlations, and in order to prevent potential negative effects to pork quality, carcass characteristics or growth the relationships between these traits must be quantified and considered in selective breeding schemes.

The main objective of this study was to estimate heritability and genetic correlations for various novel pork quality, carcass cut weight, belly, carcass leanness and growth traits in a population of purebred, terminal-line Duroc gilts.

## 2. Materials and Methods 

### 2.1. Ethics Statement

The animals included in this study were managed in accordance with the “Code of practice for the care and handling of pigs” (National Farm Animal Care Council, 2014). All the samples for genotyping were collected in a nucleus breeding farm and the animal owners agreed to be involved in the project. The slaughters, data collection and trait measurements were done by well-trained staff following industry best practices. 

### 2.2. Datasets

Phenotypic records for 39 growth, carcass, pork quality and conventional carcass traits were available for 2583 pigs (all female) born between 2010 and 2018. All animals were born and raised on the same nucleus farm and slaughter information was collected at the same slaughter plant on pigs from 159 to 219 days of age. Only female records were collected as the males were used for genetic dissemination from the nucleus farm.

The initial pedigree file contained 193,764 animals, in which 3796 were sires and 19,802 were dams. The pedigree was trimmed to 10 generations back from the phenotyped animals. The number of animals in the pedigree file ranged from 2544 to 4917 for the different traits (average ± SD: 4579 ± 689).

### 2.3. Trait Description

A total of 39 growth, conventional, carcass and pork quality traits were included in this study. The descriptive statistics are shown in Table 1 and Table 2. Traits were grouped into four categories: growth, pork quality and conventional and novel carcass traits. 

**Growth traits.** Average daily gain (ADG; g/day) was defined as total body weight gain divided by days-on-test. Pre-slaughter live weight (LW; kg) was measured five days before slaughter. Hot carcass weight (HCW; kg) was defined as the whole carcass weight taken after exsanguination and evisceration, including the head, leaf lard, kidneys and trotters [20]. 

**Pork quality traits**. Drip loss (DL) measurements were obtained following a retail method by cutting a 2.5 cm thick chop from the center of the loin and weighing it to obtain the preliminary value (DL1). Subsequently, the loin was packaged in a retail tray with a moisture pad for 48 h and weighed a second time to obtain the difference in moisture weight (DL2). Percent drip loss was calculated as the difference between DL2 and DL1 divided by DL1 [20]. Both the Japanese color scale (LJPC) [21] and NPPC color scale (LNC) [22] were used to classify the loin cuts by color. As outlined by Fortier et al. [20], the LJPC scale ranges from 0.5 to 6.0 while the LNC scale ranges from 1.0 to 6.0. Loins were also assigned a marbling score according to the NPPC marbling scale (LNM) [22] on a scale from 1 to 10, which corresponds to the indirect percentage of intramuscular lipid content. Each loin was scored a single time by technicians at the time of processing. In addition, Minolta color measurements were taken using the Minolta Colorimeter (Minolta Camera Co., LTD, Osaka, Japan), in which L* (lightness), a* (redness) and b* (yellowness) values were determined as averages of two measurements. At 24 h after slaughter, loins were measured twice with a pH meter [20] to obtain the loin ultimate pH average (LPHA). 

**Conventional carcass traits**. Conventional carcass traits were defined as traits that are commonly measured by processors or producers. Dressing percentage (DP; %) was calculated by dividing HCW by LW and was recorded as a percentage. Both backfat (GBF) and loin (GLD) depth measurements were obtained using a Destron optical probe (Destron-Fearing, Saint Paul, MN) at the third to fourth from the last rib. A grade index (GI) was measured on each carcass in order to assess economic quality of the carcass on a percentage-based scale. The GI is a grid created by packers to reward the most desirable carcasses, whether by size or lean yield. Technicians were responsible for measuring loin length (LL; cm), measured from midway between 2nd and 3rd ribs to the posterior end of the loin cut, 2.5 centimeters in front of the pelvis and ruler muscle depth (RMD; cm)—a hand measure of the diameter of the loin-eye between 3^rd^ and 4^th^ last ribs taken seven centimeters from the midline and perpendicular to the skin surface [20]. Loin area (LA; cm^2^) and loin circumference (LC; cm) were measured using the ImageJ software [23]. Loin images were processed using the ImageJ software [23] and used to validate the RMD measurement. All loin measurements were taken from the cross section of the 3rd and 4th last ribs [20]. 

**Novel carcass traits**. In this context, novel carcass traits were defined as traits that are not commonly used for genetic parameter estimation or included in selection indexes. Carcasses were separated into primal cuts to attain both untrimmed and trimmed weights. Untrimmed belly weight (UBLW; kg), untrimmed Boston butt weight (UBW; kg), untrimmed ham weight (UHW; kg), untrimmed loin weight (ULW; kg) and untrimmed shoulder weight (USW; kg) were weighed with skin, fat and bones remaining. Then, trimmed belly weight (TBLW; kg), trimmed Boston butt weight (TBW; kg), trimmed picnic shoulder weight (TSW; kg) and trimmed ham weight (THW; kg) were taken post removal skin with fat trimmed to commercial levels. A boneless loin weight (BLW; kg) was measured with skin, bone, tenderloin and sirloin removed with fat trimmed to 6.35 millimeters. Sirloin weight (SW; kg) and tenderloin weight (TW; kg) were taken after removal from the primal loin. Two sub-primal rib cuts were measured as back ribs weight (BRW; kg) and side ribs weight (SRW; kg) after extraction from the primal loin and the primal belly, respectively. In order to quantify the belly fat quality, the belly flop test (BLFT; cm) was performed by hanging the belly, skin down over a metal pipe. After two minutes, the distance between the sides of the belly was measured [20] in centimeters. In addition, belly length (BL; cm) was measured as the length of the whole belly; belly width (BLW; cm) was measured at the center of the cut; and width of the rear (BWR; cm) was measured at the end of the cut. All individual carcass measurements were taken during slaughter by a single well-trained technician per day of slaughter.

### 2.4. Definition of Statistical Models

Evaluation of fixed and random effects included in the statistical model was performed in the R software [24] using the *lm* (linear model) and *AIC* (Akaike information criterion) functions. The fixed effects tested were: slaughter technician (SLT; i.e., the technician who took the carcass measurements), slaughter date (SLD; date of animal slaughter), season-year (SY; season (1: November–January; 2: February–April; 3: May–July; 4: August–October) and year at time of birth), parity of the dam (P1vP2up; parity one dams versus parity two and greater dams, and P1vP2vP3up; parity one dams versus parity two dams versus parity three and greater dams), age (AGE; age of the animal at the time of slaughter), hot carcass weight (HCW) and dam-litter (DL; dam and birth litter information). First degree interactions were also tested, but only SLD by SLT was found to be significant (*p*-value < 0.05).

The process of backwards selection was used to remove non-significant effects one at a time per trait until each remaining effect or interaction effect was significant at an α of 0.05 and produced the lowest residual error. Additionally, the Tukey test was performed for traits significant for the parity effects to determine which parity grouping should be used. The results indicated that P1vP2up should be used as the parity effect. AIC values were used to compare models containing random effects. Those models with the lowest AIC values were chosen for further analysis. The random effects tested were DL as a common environmental (common litter) effect and animal additive genetic effects. The final models for each trait can be found in Table 3.

### 2.5. Estimation of Variance Components

Variance components were estimated using the pedigree-based relationship matrix and the restricted maximum likelihood (REML) procedure with the average-information algorithm implemented in the AIREMLF90 package [25]. Genetic correlations and their corresponding standard errors were obtained from the bivariate analysis of traits, while phenotypic correlations were obtained by adjusting the phenotypes by their fixed effects and using the Pearson correlation in the R software [24]. Heritabilities were estimated on a single-trait basis.

The animal model used when one random effect was present is:

(1) y=Xb+Zu+e

with

(2) $$\left(\begin{array}{c} u\\ e \end{array}\right)\sim N\left[\left(\begin{array}{c} 0\\ 0 \end{array}\right),\left(\begin{array}{cc} \sigma_{a}^{2} & 0\\ 0 & \sigma_{e}^{2} \end{array}\right)\right]$$

where **y** is the vector of phenotypic observation, **b** is the vector of fixed effects (found to be significant for each trait), **u** is the vector of additive genetic effects and **e** is a vector of random error. The **X** and **Z** are the incidence matrices associating **b** and **u** to the observations, respectively. The $\sigma_{a}^{2}$ and $\sigma_{e}^{2}$ are the additive and residual error variances, respectively.

The animal model used when both DL and animal additive genetic random effects were present is:

(3) y=Xb+Zu+Wd+e

with

(4) $$\left(\begin{array}{c} u\\ d\\ e \end{array}\right)\sim N\left[\left(\begin{array}{c} 0\\ 0\\ 0 \end{array}\right),\;\left(\begin{array}{ccc} \sigma_{a}^{2} & 0 & 0\\ 0 & \sigma_{d}^{2} & 0\\ 0 & 0 & \sigma_{e}^{2} \end{array}\right)\right]$$

where **y** is the vector of phenotypic observation, **b** is the vector of fixed effects (found to be significant for each trait), **u** is the vector of additive genetic effects, **d** is the vector of common environment (dam-litter) effect and **e** is a vector of random error. The **X, Z** and **W** are the incidence matrices associating **b, u** and **d** to the observations, respectively. $\sigma_{a}^{2}$, $\sigma_{d}^{2}$ and $\sigma_{e}^{2}$ are the additive genetic, common environmental (dam-litter) and residual error variances, respectively.

## 3. Results

### 3.1. Descriptive Statistics

The descriptive statistics of the phenotypic records are shown in Table 1 and Table 2. There are a total of 18 carcass traits including primal and subprimal cuts, 10 pork quality traits, three growth traits and eight conventionally measured traits. The average number of observations among growth traits was 2224 (±14), while for pork quality, conventional and novel carcass trait averages were 2045 (±331), 1992 (±502) and 1942 (±575), respectively. The variation in the number of observations is due to the data trimming process. The BLFT, BWR and LC had less observations (570, 572 and 777, respectively) as they were added later in the data collection process. 

On average, trimmed primal cut weights were 24.79 ± 7.01 percent less than the untrimmed cuts.

### 3.2. Statistical Models

The final models for each trait are shown in Table 3 (categorical fixed effects, covariates and random effects) and Table 4 and Table 5 (significance levels and AIC values, respectively). Fixed effects such as SLDxST and SY were used to account for variation in the slaughter process and the season and year when the animal was born, respectively. Similarly, the parity effect was used to account for the differences in performance among pigs born from gilts, as it has been shown that pigs born from earlier parity dams have lower performances [26,27,28]. 

### 3.3. Heritabilities

The heritability estimates are shown in Table 6 and Table 7. Results were categorized using the following heritability scale: low: from 0.01 to 0.14; moderate: from 0.15 to 0.39; and high: greater than or equal to 0.40. 

In general, growth and weight traits had moderate heritabilities, with values of 0.28 ± 0.07, 0.30 ± 0.06 and 0.26 ± 0.06 estimated for ADG, HCW and LW, respectively. Pork quality traits were lowly to highly heritable, ranging from 0.14 ± 0.05 (LNC) to 0.42 ± 0.06 (LNM). The Minolta color scores were moderate in magnitude with an average of 0.33. The LNC and LJPC had lower heritabilities of 0.14 ± 0.05 and 0.22 ± 0.05, respectively, and DLP (h^2^ = 0.28 ± 0.09) and LPHA (h^2^ = 0.39 ± 0.07) were moderately heritable. Conventionally-measured carcass traits were lowly to highly heritable with a range of 0.14 ± 0.05 (DP) to 0.47 ± 0.08 (LA) with the exception of GI (its heritability was estimated as 0.00 ± 0.00). This range in heritabilities was expected, as these traits have been used in swine selection for years due to their moderate to high heritabilities. Measurements taken on the loin ranged from 0.23 ± 0.09 (LC) to 0.47 ± 0.08 (LA) with an average heritability of 0.34 ± 0.10. GBF had a heritability of 0.38 ± 0.07.

The heritability estimates for novel carcass traits ranged from 0.10 ± 0.04 (BW) to 0.40 ± 0.06 (BLW) and 0.40 ± 0.07 (THW). The novel belly traits had low to moderate heritabilities with an average of 0.19 ± 0.08, while subprimal cuts ranged from low to highly heritable with an average of 0.25 ± 0.10. The heritability estimated for BLFT of 0.31 ± 0.11 was the highest among the belly traits. Trimmed and untrimmed primal cuts ranged from lowly to highly heritable. Trimmed cuts tended to have a higher heritability (average of 0.25 ± 0.11) compared to untrimmed cuts (average of 0.20 ± 0.04), which could be due to trimmed cuts having skin and some excess fat tissue removed.

### 3.4. Genetic Correlations 

The genetic correlations are shown in Table 8, Table 9, Table 10, Table 11, Table 12 and Table 13. In general, genetic correlations between conventionally-measured carcass traits and pork quality traits were moderate in magnitude with the exception of a few highly correlated traits. The genetic correlations estimated between pork quality and novel carcass traits, and between conventional and novel carcass traits, were moderate.

Among the growth and conventional carcass traits, high to moderate genetic correlations were observed, with high and favorable correlations being prominent among growth traits specifically. Similarly, genetic correlations among pork quality traits were moderate to high. Novel carcass traits tended toward a moderate degree of association with fewer trait pairs having high correlations.

Phenotypic correlations are not discussed in the paper, but for completeness they are presented as Supplementary Material.

## 4. Discussion

### 4.1. Statistical Models

For some groups of traits (i.e., growth and carcass traits), multiple covariates were found to be significant during the model development process. The final covariates fitted in the models (Table 3, Table 4 and Table 5) were chosen based on biological and industry considerations, and precedents established in the literature. For example, HCW and LW included age as a covariate, as they are measures of body weight and carcass growth. Another example was adjusting carcass traits for HCW to have the estimates be analyzed by content of lean muscle and fat instead of weight by accounting for lighter or heavier carcasses. For other traits, such as ADG, no covariate was added to the model due to potential confounding factors, as ADG was calculated directly from the age and weight of the animal. 

Other studies have used age and cold carcass weight (CCW) as covariates to estimate genetic parameters. Miar et. al [29] used age and CCW as covariates, while van Wijk et. al [16] also used CCW. Age is a common fixed effect due to older animals growing larger and naturally having greater body mass and measurement than a younger animal. CCW has been used in other studies to adjust for the size of the carcass, similar to the use of HCW in this study.

Color traits were significantly affected by both AGE and HCW in some instances. In one study [30], the effect of age on meat color was studied using the Minolta scale. Age was observed to have a significant effect on L* and b*, and one on the ratio between a* and b*, whereas a* was not significant. Other studies have reported that meat becomes redder with age due to the concentration of myoglobin [31]. Pork color has also been related to the type of muscle fiber present, as lighter meat will often have more Type IIB fibers [32]. For these reasons, both were considered valid covariates for the statistical models used in this study.

The models in this study do not include some of the typical fixed effects, as seen for estimates of genetic parameters, due to the nature of the datasets. Studies available in the literature commonly use farm, sex, herd-year-season or slaughter plant (in the case of carcass traits) to define their contemporary groups [13,15,33,34]. However, this study dealt with only female pigs that were raised on the same farms from birth to slaughter and were also slaughtered at the same slaughter plant. Therefore, our models did not consider these factors due to the lack of variability. Past literature has shown that there is a significant difference in results between the sexes in pigs [35,36,37], and as such, further study in this area should be conducted with information from boars and barrows. However, the impact in the genetic parameter estimates is expected to be minimal, as sex is usually accounted for in statistical genetic models.

### 4.2. Heritabilities

Growth, pork quality and conventional carcass trait heritabilities and variance components are presented in Table 6. Growth and weight traits had moderate heritabilities with estimates of 0.28 ± 0.07, 0.30 ± 0.06 and 0.26 ± 0.06 for ADG, HCW and LW, respectively. The heritability estimated for ADG in this study is lower than estimates reported in other studies for the same trait (e.g., 0.36 ± 0.07 and 0.47 ± 0.02) in the Duroc breed [14,38]. However, it is similar to estimates found for other breeds: 0.24 (no SE presented) in Large White [39] and 0.27 ± 0.03 in Landrace [40] pigs. The estimate of 0.30 ± 0.06 for HCW falls within the range seen in the literature (0.24 – 0.36) [41,42,43]. The heritability estimate for LW is in agreement with the body weight by age heritability curve presented by Edwards et al. [44]. The heritability and additive genetic variance estimates indicate that growth traits are under moderate genetic control and can be improved through direct genetic selection. For instance, high genetic progress for growth traits has been reported in various pig populations [45,46,47].

Pork quality traits had moderate heritabilities with the only low heritability estimated for LNC (0.14 ± 0.05). To the best of our knowledge, a heritability for color score based on the NPPC scale has not been previously reported. There is more information available in the literature for the Japanese color scale, possibly due to the popularity of this color scale. Suzuki et al. [14] estimated a heritability of 0.18 ± 0.02 for LJPC, while this study found a slightly higher heritability of 0.22 ± 0.05. Meanwhile, another study found a heritability of 0.83 ± 0.12 [48], which is above the common range observed for meat quality traits in livestock species. 

The Minolta color scale (L*, a*, and b*) is used to quantify the color of pork based upon lightness, redness and yellowness values given by a machine. The heritability estimate for L* (0.36 ± 0.07) is within the range of 0.15−0.57 [5, p 358] reported in literature, though it is greater than the value of 0.16 ± 0.02 estimated in another Duroc population [14]. The heritability estimated for a* (0.30 ± 0.06) is similar to that estimated in a crossbred commercial population (0.36 ± 0.06) [13], but is different from another estimate of 0.52 ± 0.10 [40]. Our estimate of b* (0.32 ± 0.06) is different from those found in literature, as it is higher than 0.20 ± 0.06 found by Miar et al. [13] and less than the 0.94 ± 0.11 found by Newcom et al. [48]. However, Newcom et al. [48] stated that the high heritability estimates in their study could have been due to the study design and limited environmental variation.

Pork color scales generally had lower heritability (average of 0.18) compared to the Minolta color measurements (average of 0.33). This difference could be due to human error, as color scores were given by a technician while Minolta L*, a* and b* were measurements taken using a machine. Thus, these measurements would be expected to have less error.

The DLP heritability found here (0.28 ± 0.09) is within the range of what was found in literature [49], though it is higher than the heritability of 0.14 ± 0.01 estimated by Suzuki et al. [14] with a Japanese population of Duroc pigs. The main factors that influence DLP are the rate of pH decline postmortem and the ultimate pH of the meat. Additionally, sarcomere length and other environmental factors may also influence the amount of drip loss from a pork product [50].

Previous heritability estimates for LNM range from 0.16 ± 0.07 to 0.23 ± 0.05 to 0.31 ± 0.12 [13,16,38], which are lower than the estimated 0.42 ± 0.06 found in this study. Heritability for LPHA has a range from 0.07 to 0.39 ([9] p. 358); the estimate in this study (0.39 ± 0.07) is among the higher estimates. The higher heritabilities found for these traits could be due to the amount of variation in this population of Duroc pigs, as it is comprised of a combination of purebred animals from Europe, Canada and the United States. In addition, there are other factors that influence the heritability estimates, including the statistical method used, variables included in the models, sample size and trait recording.

Conventional carcass traits were generally moderately to highly heritable, with the exception of GI, which had an estimated heritability of 0.00 ± 0.00. This indicates that GI is not under genetic control, as grades are determined by individual packing plants and fluctuate based on plant and time of the year, making genetic prediction difficult. Therefore, GI (as currently measured) is not a trait that should be included in a genetic or genomic evaluation scheme.

A heritability of 0.14 ± 0.05 was found for DP in this study as compared to estimates of 0.32 ± 0.04, 0.40 ± 0.03 and 0.31 ± 0.06 for maternal breeds (Landrace, Large White Sire and Large White Dam, respectively) [51]. In another study on the Duroc breed, a heritability of 0.22 (no SE presented) was found [52], which is similar to our estimate, indicating that heritability for dressing percentage in terminal lines is lower than in maternal lines. Additionally, the lower heritability observed in this study could be due to the delay in collecting live weight to carcass weight data, as live weight was measured three days prior to slaughter.

A heritability of 0.38 ± 0.07 was estimated for GBF, which is below the average of the range shown in Clutter [53]: 0.12 – 0.74. In another population of Duroc pigs, the heritability for backfat was 0.65 ± 0.06 [54], while in a population of Berkshire pigs it was 0.57 ± 0.06 [49]. On the other hand, Miar et al. [13] found a heritability of 0.31 ± 0.06 in a population of crossbred pigs. GLD had a heritability of 0.27 ± 0.06, which falls within the range (0.13 ± 0.06 to 0.41 ± 0.06) estimated by Van Wijk et al. [16] and Miar et al. [13]. Similarly, our estimate for LA (0.47 ± 0.08) is average compared to estimates (0.22 to 0.80) found by Miar et al. [13] and Lo et al. [38], respectively, and is similar to another estimate in a population of Duroc pigs of 0.45 ± 0.02 [14]. The heritability for LL was estimated to be 0.32 ± 0.06, which is slightly lower than another estimate of 0.46 ± 0.09 [55] reported in Large White pigs, but is close to an estimate of 0.39 (no SE presented) in Landrace pigs [56]. Specific heritability estimates for LC (0.23 ± 0.09) and RMD (0.39 ± 0.07) are scarce, though they can be related to other measurements of the loin, such as LA, which indicate the size of the loin muscle.

The moderate to high heritabilities estimated for traits such as GBF, LA and RMD indicate that substantial progress can be made by selecting for these traits. Due to their higher heritabilities, genetic progress will be faster while also providing more accurate estimated breeding values.

Novel carcass traits had low to high heritabilities (Table 7). To our knowledge, there have been few estimates of these heritabilities in the literature, especially concerning a purebred terminal line of Duroc pigs. Miar et al. [13] estimated genetic parameters for similar traits in a crossbred population (Duroc x (Landrace x Large White)) and found estimates of 0.32 ± 0.06, 0.53 ± 0.06, 0.29 ± 0.05, 0.63 ± 0.04, 0.44 ± 0.06, 0.49 ± 0.06, 0.46 ± 0.06, 0.63 ± 0.06 and 0.55 ± 0.06 for SRW, TBLW, TBW, THW, TPW, UBLW, UHW, ULW and USW, respectively, as compared to the estimates received in this study of 0.28 ± 0.06, 0.18 ± 0.06, 0.26 ± 0.05, 0.40 ± 0.07, 0.14 ± 0.05, 0.16 ± 0.05, 0.23 ± 0.06, 0.25 ± 0.06 and 0.22 ± 0.06 for the same traits. The SRW and TBW have similar estimates between the studies, while the remaining traits (TBLW, THW, TPW, UBLW, UHW, ULW and USW) had lower estimates in this study. TW was estimated to have a heritability of 0.30 ± 0.06, which is similar to the estimate of 0.29 ± 0.11 found by Van Wijk et al. [16]. Neither BL (0.19 ± 0.08) nor BW (0.10 ± 0.04) heritability estimates are similar to those found (0.28 ± 0.08 and 0.49 ± 0.08, respectively) by Kang et. al [57] in a population of Yorkshire pigs, which could be due to the populational (breed) difference.

To our best knowledge, the current study is the first report of heritability estimates for BLFT, BRW, BLW, BWR, SW and UBW. Thus, the heritability estimates in this study were 0.31 ± 0.11, 0.19 ± 0.05, 0.40 ± 0.06, 0.17 ± 0.12, 0.12 ± 0.05 and 0.15 ± 0.05 for these traits, respectively. 

Carcass traits had low to high heritability, with the novel and less studied traits tending towards a low to moderate heritability. These results show that these traits are under some degree of genetic control and can be used for the purpose of selecting for specific gains on the primal and subprimal cuts of the carcass. Additionally, the moderate estimate for BLFT indicates that it may be a candidate for consideration to account for belly quality in a selection index.

### 4.3. Genetic Correlations Between Growth and Conventional Carcass and Pork Quality Traits

Genetic correlations between conventional carcass traits and pork quality traits can be found in Table 8. In general, genetic correlations of interest had moderate relationships with the exception of the strong and favorable relationships between DL1 and DL2 with GLD (0.91 ± 0.09 and 0.92 ± 0.09, respectively), LA (0.95 ± 0.05 and 0.96 ± 0.05, respectively), LC (0.82 ± 0.14 and 0.74 ± 0.01, respectively) and RMD (0.80 ± 0.10 and 0.82 ± 0.10, respectively). As DL1 and DL2 are weights of 2.4 cm thick cuts of the loin, these favorable correlations were expected; this group of traits is all related to loin size. Similarly, DL1 and DL2 have moderately unfavorable correlations with GBF (−0.63 ± 0.12 and −0.65 ± 0.13, respectively). As lean loin and backfat depth are known to be inversely correlated [5,58], this was an expected finding within this population. However, this relationship is favorable and has been used for decades within the swine industry to decrease backfat depth and increase leanness in swine carcasses.

Both color score measurements (LJPC and LNC) had a moderately unfavorable correlation with DP, (−0.50 ± 0.30 and −0.47 ± 0.30, respectively) which indicates that selection for an increased ratio of internal body contents to lean muscle tissue could lead to paler pork color. A similar correlation was found between LJPC and HCW (−0.36 ± 0.04). However, Miar et al. [13] found that the Japanese color scale was lowly genetically correlated with HCW. Meanwhile, another study reported that body weight did not have an impact on the loin color [59]. This indicates that there may be a genetic relationship between these traits, but phenotypically the effect may not be observed. Overall, more studies in independent populations are needed to better understand the genetic correlation between pork color and carcass weight traits.

Backfat depth had moderate correlations of 0.26 ± 0.15, 0.38 ± 0.14 and 0.37 ± 0.15 with L*, a* and b*, respectively. This indicates that selection for backfat is likely to moderately increase the Minolta color score. Inversely, GBF and LNC have a moderate and negative correlation of −0.37 ± 0.21, which indicates that selection for backfat has an inverse relationship with the NPPC color score. The NPPC color scale is based on Minolta L* values, and as NPPC score decreases, the L* value increases. As such, these correlations align.

The moderate and favorable correlation between GBF and LNM of 0.30 ± 0.11, was expected, as these traits have been reported to be positively correlated previously [9,60]. Additionally, Suzuki et. al [14] found a correlation of 0.28 ± 0.03 in another population of Duroc pigs. Similarly, LNM had a moderate and unfavorable correlation with GLD of −0.37 ± 0.13. It is commonly known that increased back fat depth will also increase the amount of marbling in a carcass [60].

### 4.4. Genetic Correlations Between Pork Quality and Novel Carcass Traits

Genetic correlations between pork quality and novel carcass traits are shown in Table 9. A moderate and inverse correlation was found between BLFT and LJPC (−0.42 ± 0.03), which may indicate an effect of fat texture on the color score given to a pork loin. If the LJPC were selected to increase, we would expect to see the distance between the ends of the BLFT decrease, indicating a softer textured fat. Another correlation of interest is between BLFT and LNM, for which a positive and moderate value of 0.32 ± 0.25 was found, which indicates that selection for greater marbling will increase the distance between ends of the belly in the belly flop test. However, this estimate had a high standard error.

Marbling score also had a moderate inverse correlation with TPW (−0.32 ± 0.15). This indicates that if intra-muscular fat is selected for, there may be higher total fat on the carcass, which will lower the lean yield elsewhere, such as on the picnic shoulder. The moderate correlations between LNM and THW and TW (−0.29 ± 0.11 and −0.26 ± 0.09, respectively) confirm this observation. In addition, several studies confirm this genetic correlation between leanness and fat, and summaries of correlations can be found in reviews by Ciobanu et al. [9] and Steward and Schinckel [60]. 

The moderate and inverse correlations between DLP and BRW, TBW and UBW (−0.56 ± 0.32, −0.48 ± 0.27 and −0.40 ± 0.17) indicate an unfavorable relationship between carcass leanness and drip loss when compared with estimates found in other studies. Suzuki et. al. [14] reported a genetic correlation between drip loss and loin area of 0.64 ± 0.05, and an estimate of −0.25 ± 0.06 between drip loss and backfat depth. If BRW, TBW and UBW are considered to have more fat, then drip loss could potentially be decreased by selecting for these carcass traits.

### 4.5. Genetic Correlations Between Growth and Conventionally-Measured and Novel Carcass Traits

Table 10 displays the genetic correlations between conventional carcass and novel carcass traits. Moderate to high correlations were found for HCW and LW and the trimmed and untrimmed primal cuts, and subprimal cut weight. Those that were high, positive and favorable (HCW by SRW (0.88 ± 0.00), TBLW (0.74 ± 0.06), UBLW (0.73 ± 0.07), UBW (0.92 ± 0.03) and ULW (0.71 ± 0.08)]) and moderate, positive and favorable (HCW by TBW (0.37 ± 0.05) and LW by SRW (0.45 ± 0.14), SW (0.32 ± 0.25), TBLW (0.52 ± 0.15), UBLW (0.63 ± 0.14), UBW (0.37 ± 0.19), ULW (0.42 ± 0.14) and USW (0.31 ± 0.17)) indicate a strong genetic correlation between HCW and LW and the growth and lean deposition process of this population. Additionally, the moderate and inverse correlation between HCW and THW (−0.38 ± 0.05) and UHW (−0.44 ± 0.13), and the moderate and positive correlation with ADG and TBLW (0.46 ± 0.18) and UBLW (0.50 ± 0.19), indicate that the belly contains more fat than the ham. 

For those traits that had HCW removed as a covariate from the analysis in order to meet convergence criteria, there were both high and moderately positive correlations with HCW (HCW by BLW (0.47 ± 0.11), BRW (0.33 ± 0.03), SW (0.53 ± 0.22) and USW (0.77 ± 0.08)). It is explainable that both primal and subprimal cuts would increase in weight if selection for increased HCW was implemented. In the study performed by Miar et al. [13], high correlations were also found between HCW and primal and subprimal cuts of the carcass, but our findings contrast concerning the ham’s correlations. However, this could be due to the difference in the statistical models used for the analysis. Miar et al. [13] used age as a covariate, where this study used HCW (unless it was removed due to non-convergence), which makes the interpretation of the results different. As Miar et al. [13] mentioned previously, more studies should be done on these traits in independent populations for further validation.

A highly favorable correlation was found between GBF and BLFT (0.99 ± 0.07), indicating a high degree of genetic similarity between these two traits. Additionally, a moderately favorable genetic correlation was found between GBF and TBLW (0.51 ± 0.16), a strong and negative correlation was estimated between GBF and THW (−0.73 ± 0.08), and a moderate and negative correlation was observed between BLFT and loin size traits GLD (−0.38 ± 0.28), LA (−0.49 ± 0.25) and LC (−0.68 ± 0.62). GBF also had moderately unfavorable correlations (average of −0.49) with most primal and subprimal cuts except TBLW, as shown above. This correlation is unfavorable because if GBF is included in a selection index, producers do not want to decrease the amount of lean product produced in the rest of the carcass. In this context, these correlations can be explained by considering the fat and lean contents the traits possess, as the literature indicates that fat content and lean content will have an inverse genetic correlation [53,60]. 

### 4.6. Genetic Correlations among Growth and Conventionally-Measured Carcass Traits

Genetic correlations among growth and conventional carcass traits can be found in Table 11. Among growth traits specifically, there are high and favorable correlations (ADG with HCW (0.93 ± 0.04) and LW (0.97 ± 0.04) and HCW by LW (0.90 ± 0.04)), indicating each of these traits could be used as a predictor for the others. In this study, ADG was calculated directly from the weight of the animal, which explains this high degree of genetic correlation. Past research found similar correlation between ADG and HCW [29,58]. Similarly, correlations measured on the loin (GLD and LA (0.90 ± 0.06), LC (0.83 ± 0.27) and RMD (0.91 ± 0.06); LA and LC (0.99 ± 0.01) and RMD (0.86 ± 0.06); RMD and LC (0.95 ± 0.31)) have high and favorable correlations. Because of this high degree of genetic correlation, it can be concluded that a similar set of genes influences this group of traits and suggests one trait could be used as a predictor for all the traits.

A moderate, but favorable correlation of 0.44 ± 0.19 was found between DP and ADG, and HCW by GI had a highly favorable correlation of 0.79 ± 0.61, indicating first that selection for increased feed gain will increase the ratio in a carcass of lean and fat to body contents, and second, that selection for a higher carcass weight will likely create a higher value product on a scale like the grade index used in this study.

### 4.7. Genetic Correlations among Pork Quality Traits

Genetic correlations amongst the pork quality traits can be found in Table 12. Drip loss, ultimate pH (pH at 24 h post mortem) and pork color have strong correlations [61,62], as when pH drops too low, pork that is pale in color, soft and squishy in texture and highly exudative (PSE) can occur. Inversely, if ultimate pH is too high, pork that is dark in color, firm in texture and dry in appearance (DFD) can occur [63]. Both are detrimental characteristics for pork quality and should be avoided. Within this population, several correlations can further define this relationship genetically. Several correlations indicate that if LPHA (−0.76 ± 0.25), LJPC (−0.74 ± 0.24) or LNC (−0.70 ± 0.13) are selected for, DLP will change in an inverse direction. For example, if the breeding goal was to increase the color score, DLP would be expected to decrease. Similarly, if selecting for an increase in L*, DLP will increase due to the high and favorable genetic correlation (0.70 ± 0.21). LPHA had comparable, albeit more moderate, correlations with L* (−0.48 ± 0.11), LJPC (0.67 ± 0.08) and LNC (0.56 ± 0.08), indicating selection for color should generally have a favorable impact upon ultimate pH. Miar et. al. [13] found similar correlations between L* and DL (0.55 ± 0.24), LPHA and DL (−0.99 ± 0.49), and LPHA and L* (−0.65 ± 0.21).

The favorable correlation between LNC and LJPC of 0.96 ± 0.00 suggests that these traits are nearly genetically identical. L* also shows a high degree of correlation with both LNC (−0.84 ± 0.13) and LJPC (−0.79 ± 0.02). Suzuki et al. [14] also found a correlation of −0.80 (no SE presented) between LJPC and L*. As the NPPC color score is based on Minolta L* values, this inverse correlation is explainable because the NPPC score decreases as L* increases, and if LNC and LJPC are genetically the same trait, then the Japanese color score should also decrease. 

Among the Minolta values, moderate to high correlations were observed. Correlations between L* and a* (0.47 ± 0.13), L* and b* (0.82 ± 0.06) and a* and b* (0.86 ± 0.05) are all positively related. Lee et al. [49] found a similar estimate for L* and b* of 0.75 (no SE presented) and a* and b* of 0.41 (no SE presented), but a lower correlation between L* and a* of 0.03 (no SE presented). Miar et al. [13] reported moderate to high correlations among the Minolta color scale (L* and a* were −0.40 ± 0.15, L* and b* were 0.51 ± 0.12, and a* and b* were 0.46 ± 0.13), but estimated an inverse correlation between L* and a*, contrary to this study. However, in general the Minolta color scale has a positive and moderate to high correlation.

Moderate correlations were found between LNM and DLP (−0.30 ± 0.19), L* (0.34 ± 0.13), a* (0.54 ± 0.12) and b* (0.45 ± 0.12). Marbling and drip loss had a low and negative correlation of −0.06 ± 0.19 as estimated previously by Miar et al. [13]; however, this estimate had high standard error. Miar et al. [13] and Khanal et al. [15] also estimated correlations between LNM and the Minolta scale (L* of −0.12 ± 0.16; a* of −0.03 ± 0.15; b* of −0.13 ± 0.17 and L* of 0.11 ± 0.30; a* of 0.02 ± 0.34; b* of 0.17 ± 0.44, respectively), but none of these estimates are similar. The differences among these estimates could be due to the different breeds of animals used in each study. Where this study worked with terminal Durocs, Miar et al. worked with commercial crossbreds and Khanal et al. worked with maternal lines. Additionally, fat does not carry any myoglobin, the primary molecule that provides pigment in meat products, so it would stand to reason that the presence of more marbling may reflect more light, giving a higher L* value [64]. However, more studies should be done to better understand the genetic correlations between marbling and the Minolta scale within the Duroc breed.

### 4.8. Genetic Correlations among Novel Carcass Traits

Genetic correlations for novel carcass traits can be found in Table 13. As would be expected due to their part-whole correlation, the trimmed and untrimmed primal cuts are highly correlated. TBLW and UBLW had a correlation of 0.87 ± 0.04; TBW and UBW had a correlation of 0.80 ± 0.05; UHW and THW had a correlation of 0.88 ± 0.03; USW and TBW had a correlation of 0.75 ± 0.10 and USW by TPW had a correlation of 0.77 ± 0.08. Another study also found positive estimates between these cuts. UHW and THW had a correlation of 0.70 ± 0.01, TBLW and UBLW had a correlation of 0.21 ± 0.03, while TBW and TPW were correlated with USW with values of 0.42 ± 0.03 and 0.47 ± 0.02 [13]. These correlations are similar in direction, though not in magnitude, and indicate that this study confirms what has been previously observed in the literature. Similarly, UBW and USW had a high and positive correlation of 0.82 ± 0.07, which was not studied by Miar et. al [13] and may be the first estimate presented between these two subprimal cuts.

The genetic correlation between trimmed and untrimmed primal cuts and subprimal cuts are most commonly moderate but have a few high correlations as well. SRW and BRW had a highly favorable correlation of 0.71 ± 0.14, indicating these two cuts can be selected together in a positive direction. To contrast, TBLW and UBLW tend to be strongly and inversely correlated with TPW (−0.77 ± 0.30 and −0.71 ± 0.37, respectively) and USW (−0.72 ± 0.18 and −0.61 ± 0.20, respectively). TBLW also shares moderately inverse correlations with TBW (−0.60 ± 0.16), THW (−0.33 ± 0.17), TW (−0.42 ± 0.17), BRW (−0.31 ± 0.24) and UBW (−0.55 ± 0.25). UBLW has similar correlations with UBW (−0.51 ± 0.26), TBW (−0.43 ± 0.18) and TW (−0.41 ± 0.18). As the belly would be expected to have more fat than lean mass, these inverse correlations are explainable. 

These estimates do not agree with what was found by Miar et al. [13] where correlations between trimmed and untrimmed primal cuts tend to be either strong or moderate and positive. This may be due to difference in breed composition or the use of different covariates in the statistical analysis. Where this study adjusted all carcass traits with HCW, Miar et al. [13] adjusted their traits by AGE instead. Therefore, the results may be interpreted slightly differently.

Meanwhile, cuts that contain more lean mass tend to have a moderate and positive relationship amongst each other. THW had positive correlations with TPW (0.37 ± 0.16), BRW (0.44 ± 0.16), and SW (0.47 ± 0.26). UHW has similar correlations with these traits as well. Among the loin traits (ULW, TW and SW) the average correlation was 0.47. TPW and TBW had a correlation of 0.40. These correlations indicate that the genetic relationship between leanness traits will be positive and favorable. Generally, these estimates are in agreement with previous estimates between leaner carcass traits [13]. 

The belly flop test was developed to determine the degree of firmness of the fat as a measurement of overall belly quality and is phenotypically related to fatty acid profile [65]. The distance measured between the ends of the belly are indicative of firmer (wider) or softer (closer) fat. Previous literature has explored the genetic correlation between the belly and fat traits, such as fat percentage in the belly, backfat depth, subcutaneous fat area and inter and intra-muscular fat content [66]. Recently, pork processors have begun using the belly flop test to test bacon fat quality in packing plants, where firmer fat is preferred for the later stages of processing of bacon. With this knowledge, the genetic definition of this trait could be a valuable asset for swine breeders as well. To our knowledge, this is the first paper reporting the genetic correlations between the belly flop test (and other belly traits) and carcass traits. 

Only THW was highly correlated with BLFT (−0.87 ± 0.06), though it is a strong and negative correlation. This indicates that selection for increased lean in the ham may decrease the distance measured between the ends of the bacon in the belly flop test, which is ultimately an undesirable impact upon the belly. Likewise, BLFT is moderately and inversely correlated with BRW (−0.36 ± 0.07), SRW (−0.58 ± 0.15), TBW (−0.47 ± 0.01), TPW (−0.58 ± 0.31), UHW (−0.49 ± 0.14) and UPW (−0.45 ± 0.31). With this unfavorable relationship, if producers consider including BLFT as a trait in a selection index, it may be important to include it alongside other carcass traits such as trimmed or untrimmed primal cuts to prevent any negative impacts upon bacon quality. 

One expected correlation found was between BL and BLFT with a correlation of −0.66 ± 0.64, meaning that a longer belly may produce a smaller distance between the ends of the belly during the belly flop test will be closer together. BW and BWR are also closely related (0.87 ± 0.83), indicating they are similar traits and may not need to be measured separately. Using the BLFT to determine quality and BW to determine the preferable width of the belly could be beneficial to selection programs that wish to meet a packer’s preferences, and these results show promise in regard to the degree of genetic correlation between belly traits while also providing a measure of caution when selecting for primal or sub-primal cuts.

## 5. Conclusions

Pork quality, conventionally-measured and novel carcass and growth traits in purebred Duroc pigs are heritable and can be improved through genetic selection. Genetic correlations between growth and conventional carcass traits and pork quality traits were predominantly low to moderate and unfavorable, while growth and conventional and novel carcass traits were mostly moderate and favorable in relation, with few high correlations. Meanwhile, among growth and conventional carcass traits, pork quality traits and novel carcass traits, there were higher and moderate correlations which tended to be favorable. An important finding in this study was that some trait measurements may be redundant due to their high and positive correlations, such as the correlations estimated between HCW, LW and ADG; between L*, LNC and LJPC; and between trimmed and untrimmed cuts. It may not be necessary to measure all of these traits or to include every color score in a selection index for example. Carcass and pork quality traits are not good predictors for one another due to their moderate correlation. Similarly, growth and conventional carcass traits may not have a large impact on pork quality traits, as many correlations were low (but also had large standard errors). However, growth and conventional and novel carcass traits had many favorable correlations and show potential for use within a selection index. The estimation of genetic parameters for belly traits is a valuable contribution of this paper as it fills a gap in knowledge and provides insight into using a measurement such as the belly flop test as a predictor for belly quality. These estimated parameters show potential for developing a selection index combining growth, pork quality and carcass traits.

## Figures and Tables

**Table 1:** Complete descriptive statistics for growth, pork quality and conventionally measured carcass traits.

Trait	Abbreviation	*n*	Mean	SD	Min.	Max.
**Growth traits**						
Average Daily Gain (g/day)	ADG	2226	670.07	49.14	518.00	813.00
Hot Carcass Weight (kg)	HCW	2237	100.61	5.92	83.60	118.00
Live Weight (kg)	LW	2210	121.17	7.18	100.00	143.00
**Pork quality traits**						
25 cm Chop Initial Weight (g) ^1^	DL1	2152	188.68	26.45	120.70	269.00
25 cm Chop Post Weight (g) ^1^	DL2	2150	186.25	26.19	114.40	261.80
Drip Loss Percentage (%)	DLP	1114	1.15	0.53	0.06	3.14
Japanese Loin Color Scale	LJPC	2203	3.54	0.38	2.50	4.50
Minolta L*	L*	2089	49.22	2.30	41.80	57.40
Minolta a*	a*	2092	4.17	1.64	0.25	17.85
Minolta b*	b*	2082	9.12	1.07	5.70	12.50
NPPC Loin Color Scale	LNC	2202	3.60	0.50	2.50	5.00
NPPC Loin Marbling Scale	LNM	2209	2.11	0.67	1.00	4.00
Loin pH	LPHA	2155	5.72	0.15	5.25	6.18
**Conventionally measured carcass traits**						
Dressing Percentage (%)	DP	2159	83.13	2.77	74.73	91.90
Grading Back Fat (cm)	GBF	2218	14.16	3.13	7.10	24.10
Grade Index ^2^	GI	2216	113.07	4.18	70.00	115.00
Grading Loin Depth (cm)	GLD	2224	66.62	5.41	51.00	82.40
Loin Area (cm^2^)	LA	1921	56.90	7.04	31.94	80.41
Loin Circumference (cm)	LC	777	29.78	2.51	21.22	53.55
Loin Length (cm)	LL	2209	67.46	2.52	60.00	75.00
Ruler Muscle Depth (cm)	RMD	2215	70.87	5.12	55.00	85.00

^1^ Initial chop weight was the weight of the 25 cm loin sample taken prior to packaging, while post chop weight was the weight of the 25 cm loin sample taken after the loin was packaged for 48 h. ^2^ Grade index is a measure of the economic value of each carcass based upon lean content and fat content.

**Table 2:** Complete descriptive statistics for novel carcass traits.

Trait	Abbreviation	*n*	Mean	SD	Min.	Max.
Belly Flop Test (cm)	BLFT	570	15.21	4.50	3.00	29.50
Belly Length (cm)	BL	1036	62.93	2.92	54.00	72.00
Boneless Loin Weight (kg)	BLW	2221	4.83	0.45	3.52	6.18
Back Ribs Weight (kg)	BRW	2211	0.90	0.13	0.50	1.28
Belly Width (cm)	BW	1824	26.67	1.84	13.00	34.00
Belly Width Rear (cm)	BWR	572	19.05	2.36	11.00	26.00
Side Ribs Weight (kg)	SRW	2190	1.75	0.23	1.09	2.42
Sirloin Weight (kg)	SW	2206	1.12	0.19	0.58	1.73
Tenderloin Weight (kg)	TW	2212	0.49	0.08	0.28	0.74
Trimmed Belly Weight (kg)	TBLW	2181	5.87	0.63	3.96	7.80
Trimmed Boston Butt Weight (kg)	TBW	2222	4.69	0.59	3.04	6.34
Trimmed Ham Weight (kg)	THW	2204	9.42	0.71	7.33	11.60
Trimmed Picnic Shoulder Weight (kg)	TPW	2219	4.18	0.48	2.74	5.54
Untrimmed Belly Weight (kg)	UBLW	2211	7.63	0.75	5.50	9.84
Untrimmed Boston Butt Weight (kg)	UBW	2224	5.65	0.62	4.00	7.34
Untrimmed Ham Weight (kg)	UHW	2219	11.42	0.78	9.12	13.80
Untrimmed Shoulder Weight (kg)	USW	2216	11.71	0.89	9.04	14.38
Untrimmed Loin Weight (kg)	ULW	2223	11.18	0.83	8.69	13.72

**Table 3:** Fixed and random effects included in the statistical models used for the single and two-trait analysis.

Traits ^1^	Fixed Effect(s) ^2^	Covariate(s) ^3^	Random Effect(s) ^4^
DP	SLDxST	Age	Animal
DLP, b*, LPHA	SLDxST	Age	Animal, DL
GI	SLDxST	HCW	Animal
BRW, BW, GLD, RMD, BLFT	SLDxST	HCW	Animal, DL
LNC	SLDxST, P1vP2up		Animal, DL
a*, L*, LNM	SLDxST, P1vP2up	Age	Animal, DL
LJPC	SLDxST, P1vP2up	HCW	Animal
DL1, DL2, GBF	SLDxST, P1vP2up	HCW	Animal, DL
HCW, LW	SLDxST, SY	Age	Animal, DL
BWR	SLDxST, SY	HCW	Animal
BL, LL, SRW, TBLW, TPW, TW, UBLW, UBW, ULW, USW	SLDxST, SY	HCW	Animal, DL
BLW, LA, LC, SW, TBW, THW, UHW	SLDxST, SY, P1vP2up	HCW	Animal, DL
ADG	SLDxST, SY		Animal, DL

^1^ DP: dressing percentage; DLP: drip loss percentage; b*: loin Minolta b* score; LPHA: loin pH average; GI: grade index; BRW: back ribs weight; BW: belly width; GLD: grading loin depth; RMD: ruler muscle depth; BLFT: belly flop test; LNC: loin NPPC color score; a*: loin Minolta a* score; L*: loin Minolta L* score; LNM: loin NPPC marbling score; LJPC: loin Japanese color score; DL1: drip loss measurement 1; DL2: drip loss measurement 2; GBF: grading back fat; HCW: hot carcass weight; LW: live weight; BWR: belly width at the rear; BL: belly length; LL: loin length; SRW: side ribs weight; TBLW: trimmed belly weight; TPW: trimmed picnic shoulder weight; TW: tenderloin weight; UBLW: untrimmed belly weight; UBW: untrimmed Boston butt weight; ULW: untrimmed loin weight; USW: untrimmed picnic shoulder weight; BLW: boneless loin weight; LA: loin-eye area; LC: loin-eye circumference; SW: sirloin weight; TBW: trimmed Boston butt weigh; THW: trimmed ham weight; UHW: untrimmed ham weight; ADG: average daily gain. ^2^ SLDxST: date of animal slaughter by the slaughter technician; P1vP2up: grouping of parity one dams versus parity two and greater dams; SY: season and year of animal birth. ^3^ Age: age of animal at time of slaughter; HCW: hot carcass weight of animal at time of slaughter. ^4^ DL: effect of dam and birth litter; common environmental factor.

**Table 4:** Significance of covariate and categorical fixed effects tested for growth, pork quality and conventionally measured carcass traits; and the different AIC values obtained among the models with and without the random effect of dam-litter (DL).

	Fixed Effects ^1^					Random Effects ^2^	
Trait ^3^	SLDxST	P1vP2up	SY	Age	HCW	DL	Animal + DL
**Growth traits**							
ADG	****		****			21,550.43	−135,190.70
HCW	****		****			13,603.34	−149,532.60
LW	****		****	**		14,047.96	−141,882.40
**Pork quality traits**							
DL1	****	**			****	18,696.57	−127,322.10
DL2	****	**			****	18,612.74	−128,274.10
DLP	****			***		1059.23	-Inf
LJPC	****	***				-	−160,829.40
L*	****	**		*		8359.99	−160,829.40
a*	****	**		***		6159.43	−142,888.80
b*	****			***		5031.52	−144,782.00
LNC	****	*				1321.70	−156,996.80
LNM	****	**		*		2795.43	−145,330.40
LPHA	****			**		−4144.23	−155,993.50
**Conventionally measured carcass traits**							
DP	****			**		-	−163,952.10
GBF	****	**	*		****	9988.46	−147,518.00
GI	****				****	-	−159,068.30
GLD	****				****	12,787.05	−138,065.40
LA	****	**	*		****	11,061.23	−122,852.30
LC	****	**	**		****	2630.98	−42,713.54
LL	****		**		****	8653.23	−138,682.60
RMD	****				****	12,476.45	−139,640.40

**** < 0.0001, *** < 0.001, ** < 0.01, * < 0.05. ^1^ SLDxST: date of animal slaughter by the slaughter technician; P1vP2up: grouping of parity one dams versus parity two and greater dams; SY: season and year of animal birth; Age: age of animal at time of slaughter; HCW: hot carcass weight of animal at time of slaughter. ^2^ DL: effect of dam and birth litter; common environmental factor. ^3^ ADG: average daily gain; HCW: hot carcass weight; LW: live weight; DL1: 25 cm chop initial weight; DL2: 25 cm chop post weight; DLP: drip loss percentage; LJPC: Japanese loin color scale; L*: Minolta L*; a*: Minolta a*; b*: Minolta b*; LNC: NPPC loin color scale; LNM: NPPC loin marbling scale; LPHA: loin pH; DP: dressing percentage; GBF: backfat depth; LA: loin area; LC: loin circumference; LL: loin length; RMD: ruler muscle depth.

**Table 5:** Significance of covariate and categorical fixed effects tested for novel carcass traits; and the different AIC values obtained among the models with and without the random effect of dam-litter (DL).

	Fixed Effects ^1^					Random Effects ^2^	
Trait ^3^	SLDxST	Parity	SY	Age	HCW	DL	Animal + DL
BLFT	****				**	2440.94	−35,504.03
BL	****		*		****	4036.71	−55,987.57
BLW	****	***	****		****	802.40	−153,389.30
BRW	****				****	−4818.93	−159,264.60
BW	****				****	-	−129,307.10
BWR	****		**		****	-	−37,851.27
SRW	****		***		****	−2727.63	−152,472.80
SW	****	*	*		****	−2189.68	−146,506.50
TW	****		*		****	−7239.72	−156,483.90
TBLW	****		****		****	1720.85	−150,733.80
TBW	****	*	****		****	845.88	−145,625.50
THW	****	**	****		****	2967.29	−143,913.60
TPW	****		*		****	563.55	−146,284.40
UBLW	****		***		****	2090.03	−150,757.10
UBW	****		**		****	1056.23	−146,604.20
UHW	****	**	****		****	845.88	−141,628.50
USW	****		****		****	2167.23	−143,398.00
ULW	****		****		****	3070.93	−146,928.40

**** < 0.0001, *** < 0.001, ** < 0.01, * < 0.05. ^1^ SLDxST: date of animal slaughter by the slaughter technician; P1vP2up: grouping of parity one dams versus parity two and greater dams; SY: season and year of animal birth; Age: age of animal at time of slaughter; HCW: hot carcass weight of animal at time of slaughter. ^2^ DL: effect of dam and birth litter; common environmental factor. ^3^ BLFT: belly flop test; BL: belly length; BLW: boneless loin weight; BRW: back ribs weight; BW: belly width; BWR: belly width rear; SRW: side ribs weight; SW: sirloin weight; TW: tenderloin weight; TBLW: trimmed belly weight; TBW: trimmed Boston butt weight; THW: trimmed ham weight; TPW: trimmed picnic shoulder weight; UBLW: untrimmed belly weight; UBW: untrimmed Boston butt weight; UHW: untrimmed ham weight; USW: untrimmed shoulder weight; ULW: untrimmed loin weight.

**Table 6:** Estimates of heritability (h^2^), additive genetic variance (σ^2^_a_) and variance for the permanent environmental effect of dam-litter (σ^2^_d_) for growth, pork quality and conventionally measured carcass traits.

Trait ^1^	h^2^	σ^2^_a_	σ^2^_d_	c^2^
**Growth traits**				
**ADG**	0.28 ± 0.07	522.490	416.2300	0.223
**HCW**	0.30 ± 0.06	8.442	0.1030	0.004
**LW**	0.26 ± 0.06	10.337	1.1060	0.028
**Pork quality traits**				
**DL1**	0.24 ± 0.06	124.740	63.6810	0.123
**DL2**	0.23 ± 0.06	115.630	60.3760	0.120
**DLP**	0.28 ± 0.09	0.052	0.0054	0.000
**LJPC**	0.22 ± 0.05	0.023		
L*	0.36 ± 0.07	1.310	0.0324	0.009
a*	0.30 ± 0.06	0.392	0.1320	0.101
b*	0.32 ± 0.06	0.224	0.0005	0.001
**LNC**	0.14 ± 0.05	0.015	0.0029	0.026
**LNM**	0.42 ± 0.06	0.097	0.0000	0.000
**LPHA**	0.39 ± 0.07	0.004	0.0007	0.069
**Conventionally measured carcass traits**				
**DP**	0.14 ± 0.05	0.627		
**GBF**	0.38 ± 0.07	2.852	0.4542	0.061
**GI**	0.00 ± 0.00	0.000		
**GLD**	0.27 ± 0.06	6.308	0.3462	0.015
**LA**	0.47 ± 0.08	14.042	0.4351	0.015
**LC**	0.23 ± 0.09	0.616	0.0000	0.000
**LL**	0.32 ± 0.06	1.220	0.2395	0.063
**RMD**	0.39 ± 0.07	8.245	0.3323	0.016

^1^ ADG: average daily gain; HCW: hot carcass weight; LW: live weight; DL1: 25 cm chop initial weight; DL2: 25 cm chop post weight; DLP: drip loss percentage; LJPC: Japanese loin color scale; L*: Minolta L*; a*: Minolta a*; b*: Minolta b*; LNC: NPPC loin color scale; LNM: NPPC loin marbling scale; LPHA: loin pH; DP: dressing percentage; GBF: backfat depth; LA: loin area; LC: loin circumference; LL: loin length; RMD: ruler muscle depth.

**Table 7:** Estimates of heritability (h^2^), additive genetic variance (σ^2^_a_) and variance for the permanent environmental effect of dam-litter (σ^2^_d_) for novel carcass traits.

Trait ^1^	h^2^	σ^2^_a_	σ^2^_d_	c^2^
BLFT	0.31 ± 0.11	2.950	0.0000	0.000
BL	0.19 ± 0.08	0.889	0.7091	0.152
BLW	0.40 ± 0.06	0.039	0.0000	0.000
BRW	0.19 ± 0.05	0.001	0.0003	0.034
BW	0.10 ± 0.04	0.197		
BWR	0.17 ± 0.12	0.722	0.1186	0.028
SRW	0.28 ± 0.06	0.006	0.0005	0.022
SW	0.12 ± 0.05	0.003	0.0012	0.053
TW	0.30 ± 0.06	0.001	0.0001	0.047
TBLW	0.18 ± 0.06	0.031	0.0177	0.104
TBW	0.26 ± 0.05	0.025	0.0000	0.000
THW	0.40 ± 0.07	0.111	0.0067	0.024
TPW	0.14 ± 0.05	0.013	0.0038	0.042
UBLW	0.16 ± 0.05	0.033	0.0184	0.090
UBW	0.15 ± 0.05	0.016	0.0014	0.013
UHW	0.23 ± 0.06	0.058	0.0092	0.036
USW	0.22 ± 0.06	0.048	0.0209	0.028
ULW	0.25 ± 0.06	0.072	0.0081	0.095

^1^ BLFT: belly flop test; BL: belly length; BLW: boneless loin weight; BRW: back ribs weight; BW: belly width; BWR: belly width rear; SRW: side ribs weight; SW: sirloin weight; TW: tenderloin weight; TBLW: trimmed belly weight; TBW: trimmed Boston butt weight; THW: trimmed ham weight; TPW: trimmed picnic shoulder weight; UBLW: untrimmed belly weight; UBW: untrimmed Boston butt weight; UHW: untrimmed ham weight; USW: untrimmed shoulder weight; ULW: untrimmed loin weight.

**Table 8:** Genetic correlations between conventionally measured carcass traits and pork quality traits.

Trait ^1^	DL1	DL2	DLP	LJPC	L*	a*	b*	LNC	LNM	LPHA
**ADG**	0.18 ± 0.18	−0.14 ± 0.19	−0.12 ± 0.30	−0.18 ± 0.18	−0.05 ± 0.17	−0.02 ± 0.17	0.01 ± 0.16	−0.10 ± 0.23	0.04 ± 0.14	0.11 ± 0.16
**HCW**	−0.48 ± 0.13	−0.46 ± 0.14	−0.01 ± 0.26	−0.36 ± 0.04	−0.06 ± 0.15	−0.13 ± 0.16	−0.04 ± 0.14	−0.14 ± 0.41	0.07 ± 0.13	0.05 ± 0.05
**LW**	−0.12 ± 0.19	−0.11 ± 0.19	−0.02 ± 0.25	−0.28 ± 0.04	−0.08 ± 0.17	−0.18 ± 0.17	−0.11 ± 0.14	−0.08 ± 0.23	0.13 ± 0.14	0.18 ± 0.16
**DP**	−0.12 ± 0.22	−0.10 ± 0.23	0.26 ± 0.34	−0.50 ± 0.30	0.18 ± 0.21	−0.06 ± 0.20	0.10 ± 0.05	−0.47 ± 0.30	−0.09 ± 0.05	N/A
**GBF**	−0.63 ± 0.12	−0.65 ± 0.13	0.14 ± 0.25	−0.12 ± 0.04	0.26 ± 0.15	0.38 ± 0.14	0.37 ± 0.15	−0.37 ± 0.21	0.30 ± 0.11	−0.14 ± 0.13
**GI**	0.14 ± 0.09	0.13 ± 0.10	0.42 ± 0.06	−0.05 ± 0.48	0.17 ± 0.13	0.12 ± 0.04	0.12 ± 0.60	−0.05 ± 0.09	0.10 ± 0.42	−0.15 ± 0.05
**GLD**	0.91 ± 0.09	0.92 ± 0.09	0.06 ± 0.26	−0.11 ± 0.04	0.08 ± 0.16	−0.04 ± 0.16	0.06 ± 0.15	−0.07 ± 0.22	−0.37 ± 0.13	−0.23 ± 0.15
**LA**	0.95 ± 0.05	0.96 ± 0.05	−0.07 ± 0.15	−0.09 ± 0.04	0.13 ± 0.13	0.08 ± 0.15	0.12 ± 0.14	0.03 ± 0.20	−0.15 ± 0.12	−0.17 ± 0.12
**LC**	0.82 ± 0.14	0.74 ± 0.01	−0.98 ± 0.08	0.14 ± 0.07	−0.10 ± 0.27	0.04 ± 0.27	−0.05 ± 0.26	0.09 ± 0.49	−0.26 ± 0.23	0.13 ± 0.18
**LL**	0.24 ± 0.17	0.21 ± 0.18	0.13 ± 0.22	0.18 ± 0.04	−0.09 ± 0.15	−0.04 ± 0.16	−0.12 ± 0.15	0.41 ± 0.23	−0.06 ± 0.13	0.15 ± 0.15
**RMD**	0.80 ± 0.10	0.82 ± 0.10	0.06 ± 0.24	−0.11 ± 0.15	0.16 ± 0.14	−0.05 ± 0.15	0.13 ± 0.14	−0.19 ± 0.20	−0.12 ± 0.12	−0.14 ± 0.14

N/A represents when convergence was unable to be achieved. ^1^ DL1: 25 cm chop initial weight; DL2: 25 cm chop post weight; DLP: drip loss percentage; LJPC: Japanese loin color scale; L*: Minolta L*; a*: Minolta a*; b*: Minolta b*; LNC: NPPC loin color scale; LNM: NPPC loin marbling scale; LPHA: loin pH; ADG: average daily gain; HCW: hot carcass weight; LW: live weight; DP: dressing percentage; GBF: backfat depth; LA: loin area; LC: loin circumference; LL: loin length; RMD: ruler muscle depth.

**Table 9:** Genetic correlations between pork quality and novel carcass traits.

Trait ^1^	DL1	DL2	DLP	LJPC	L*	a*	b*	LNC	LNM	LPHA
**BLFT**	−0.50 ± 0.39	−0.48 ± 0.31	0.12 ± 0.07	−0.42 ± 0.03	0.15 ± 0.28	−0.14 ± 0.24	0.02 ± 0.26	−0.47 ± 0.51	0.32 ± 0.25	0.02 ± 0.31
**BL**	0.48 ± 0.52	0.51 ± 0.55	−0.14 ± 0.19	0.28 ± 0.40	−0.06 ± 0.32	0.23 ± 0.41	0.10 ± 0.30	0.17 ± 0.48	0.14 ± 0.25	0.23 ± 0.30
**BLW**	0.99 ± 0.00	0.99 ± 0.00	0.01 ± 0.35	−0.07 ± 0.14	0.12 ± 0.13	−0.02 ± 0.14	0.08 ± 0.13	0.15 ± 0.22	−0.21 ± 0.12	−0.17 ± 0.05
**BRW**	0.25 ± 0.21	0.26 ± 0.22	−0.56 ± 0.32	0.07 ± 0.20	−0.01 ± 0.16	−0.10 ± 0.19	−0.19 ± 0.15	−0.09 ± 0.27	0.09 ± 0.08	0.36 ± 0.18
**BW**	0.37 ± 0.36	0.34 ± 0.43	0.61 ± 0.96	−0.24 ± 0.06	−0.23 ± 0.28	−0.15 ± 0.28	−0.19 ± 0.32	−0.19 ± 0.34	−0.52 ± 0.37	−0.39 ± 0.16
**BWR**	−0.32 ± 0.06	−0.36 ± 0.06	0.44 ± 0.06	0.01 ± 0.46	−0.38 ± 0.15	0.03 ± 0.03	−0.22 ± 0.38	0.01 ± 0.09	−0.39 ± 0.33	0.03 ± 0.05
**SRW**	0.36 ± 0.17	0.34 ± 0.17	0.26 ± 0.27	−0.30 ± 0.16	0.07 ± 0.15	−0.21 ± 0.16	−0.14 ± 0.13	−0.22 ± 0.25	−0.09 ± 0.09	0.12 ± 0.15
**SW**	0.59 ± 0.27	0.62 ± 0.27	−0.26 ± 0.38	−0.14 ± 0.00	−0.04 ± 0.17	−0.37 ± 0.36	−0.19 ± 0.17	−0.05 ± 0.17	−0.16 ± 0.09	0.07 ± 0.23
**TBLW**	−0.17 ± 0.22	−0.17 ± 0.22	0.10 ± 0.34	0.20 ± 0.04	−0.07 ± 0.19	0.34 ± 0.20	0.16 ± 0.16	0.08 ± 0.28	0.18 ± 0.16	−0.17 ± 0.19
**TBW**	0.08 ± 0.17	0.06 ± 0.18	−0.48 ± 0.27	0.24 ± 0.16	−0.09 ± 0.15	−0.26 ± 0.16	−0.34 ± 0.15	0.12 ± 0.21	−0.18 ± 0.14	0.29 ± 0.07
**THW**	0.72 ± 0.10	0.74 ± 0.10	0.02 ± 0.24	−0.11 ± 0.05	−0.03 ± 0.14	−0.33 ± 0.14	−0.22 ± 0.13	0.07 ± 0.21	−0.29 ± 0.11	−0.14 ± 0.13
**TPW**	0.21 ± 0.23	0.22 ± 0.23	0.12 ± 0.33	−0.21 ± 0.23	−0.09 ± 0.18	−0.26 ± 0.26	−0.13 ± 0.11	−0.10 ± 0.30	−0.32 ± 0.15	0.28 ± 0.23
**TW**	0.39 ± 0.16	0.39 ± 0.16	0.24 ± 0.29	0.03 ± 0.16	−0.11 ± 0.14	−0.27 ± 0.16	−0.24 ± 0.13	−0.09 ± 0.22	−0.26 ± 0.09	0.05 ± 0.15
**UBLW**	0.00 ± 0.24	0.00 ± 0.24	0.28 ± 0.33	0.01 ± 0.04	0.08 ± 0.20	0.25 ± 0.20	0.17 ± 0.17	−0.07 ± 0.30	0.15 ± 0.16	−0.08 ± 0.19
**UBW**	−0.33 ± 0.22	−0.34 ± 0.23	−0.40 ± 0.17	0.21 ± 0.08	−0.17 ± 0.15	−0.38 ± 0.24	−0.41 ± 0.20	−0.08 ± 0.22	0.03 ± 0.15	0.41 ± 0.19
**UHW**	0.52 ± 0.15	0.55 ± 0.15	0.02 ± 0.27	−0.15 ± 0.04	0.12 ± 0.15	−0.19 ± 0.16	−0.08 ± 0.12	−0.12 ± 0.23	−0.10 ± 0.13	−0.19 ± 0.15
**ULW**	0.64 ± 0.14	0.65 ± 0.14	0.21 ± 0.22	−0.22 ± 0.06	0.25 ± 0.15	0.06 ± 0.16	0.17 ± 0.15	−0.39 ± 0.17	−0.09 ± 0.14	−0.25 ± 0.14
**USW**	0.02 ± 0.20	0.01 ± 0.20	−0.22 ± 0.28	−0.06 ± 0.04	−0.14 ± 0.17	−0.52 ± 0.19	−0.44 ± 0.11	−0.24 ± 0.24	−0.29 ± 0.14	0.44 ± 0.16

^1^ DL1: 25 cm chop initial weight; DL2: 25 cm chop post weight; DLP: drip loss percentage; LJPC: Japanese loin color scale; L*: Minolta L*; a*: Minolta a*; b*: Minolta b*; LNC: NPPC loin color scale; LNM: NPPC loin marbling scale; LPHA: loin pH; BLFT: belly flop test; BL: belly length; BLW: boneless loin weight; BRW: back ribs weight; BW: belly width; BWR: belly width rear; SRW: side ribs weight; SW: sirloin weight; TW: tenderloin weight; TBLW: trimmed belly weight; TBW: trimmed Boston butt weight; THW: trimmed ham weight; TPW: trimmed picnic shoulder weight; UBLW: untrimmed belly weight; UBW: untrimmed Boston butt weight; UHW: untrimmed ham weight; USW: untrimmed shoulder weight; ULW: untrimmed loin weight.

**Table 10:** Genetic correlations between conventionally measured carcass traits and novel carcass traits.

Trait ^1^	ADG	HCW	LW	DP	GBF	GI	GLD	LA	LC	LL	RMD
**BLFT**	−0.00 ± 0.29	0.31 ± 0.25	0.09 ± 0.28	−0.29 ± 0.05	0.99 ± 0.07	−0.14 ± 0.70	−0.38 ± 0.28	−0.49 ± 0.25	−0.68 ± 0.62	−0.02 ± 0.26	−0.15 ± 0.26
**BL**	0.02 ± 0.38	0.71 ± 0.01	0.23 ± 0.34	−0.20 ± 0.57	−0.74 ± 0.33	0.52 ± 0.06	−0.26 ± 0.35	0.03 ± 0.30	−0.07 ± 0.61	0.97 ± 0.59	−0.08 ± 0.27
**BLW**	−0.22 ± 0.14	0.47 ± 0.11 **	0.11 ± 0.14	0.01 ± 0.05	−0.45 ± 0.11	0.12 ± 0.56	0.77 ± 0.08	0.79 ± 0.06	0.88 ± 0.18	0.21 ± 0.13	0.72 ± 0.07
**BRW**	−0.02 ± 0.20	0.33 ± 0.03 **	0.07 ± 0.20	N/A	−0.75 ± 0.13	0.22 ± 0.07	0.06 ± 0.19	0.19 ± 0.17	0.10 ± 0.09	0.54 ± 0.17	0.11 ± 0.17
**BW**	0.26 ± 0.22	0.13 ± 0.30	0.56 ± 0.30	−0.25 ± 0.05	0.01 ± 0.39	0.05 ± 0.83	−0.02 ± 0.29	0.08 ± 0.26	0.00 ± 0.45	−0.28 ± 0.31	0.13 ± 0.27
**BWR**	0.24 ± 0.06	−0.84 ± 0.02	0.46 ± 0.06	−0.38 ± 0.59	0.12 ± 0.06	N/A	−0.39 ± 0.07	−0.56 ± 0.06	−0.47 ± 0.62	0.11 ± 0.05	−0.41 ± 0.57
**SRW**	0.14 ± 0.17	0.88 ± 0.00	0.45 ± 0.14	−0.33 ± 0.19	−0.67 ± 0.11	0.79 ± 0.18	−0.17 ± 0.16	−0.10 ± 0.14	−0.16 ± 0.20	0.70 ± 0.13	−0.15 ± 0.15
**SW**	0.23 ± 0.40	0.53 ± 0.22 **	0.32 ± 0.25	N/A	−0.28 ± 0.23	0.22 ± 0.19	0.59 ± 0.27	0.62 ± 0.22	0.71 ± 0.04	−0.44 ± 0.30	0.76 ± 0.36
**TBLW**	0.46 ± 0.18	0.74 ± 0.06	0.52 ± 0.15	0.14 ± 0.25	0.51 ± 0.16	0.03 ± 0.04	−0.37 ± 0.17	−0.17 ± 0.18	−0.32 ± 0.10	−0.15 ± 0.20	−0.17 ± 0.17
**TBW**	−0.02 ± 0.17	0.37 ± 0.05	0.08 ± 0.17	−0.22 ± 0.05	−0.43 ± 0.13	0.15 ± 0.50	0.03 ± 0.16	0.06 ± 0.14	−0.04 ± 0.28	0.06 ± 0.16	−0.01 ± 0.15
**THW**	−0.12 ± 0.15	−0.38 ± 0.05	−0.01 ± 0.16	0.05 ± 0.19	−0.73 ± 0.08	0.29 ± 0.08	0.45 ± 0.13	0.54 ± 0.10	0.56 ± 0.26	0.16 ± 0.13	0.47 ± 0.11
**TPW**	0.18 ± 0.26	−0.11 ± 0.15	0.20 ± 0.22	0.12 ± 0.30	−0.39 ± 0.21	0.11 ± 0.07	0.25 ± 0.22	0.37 ± 0.19	0.64 ± 0.28	0.07 ± 0.22	0.26 ± 0.18
**TW**	−0.26 ± 0.17	0.15 ± 0.16 **	0.08 ± 0.17	N/A	−0.45 ± 0.13	0.14 ± 0.07	0.46 ± 0.14	0.43 ± 0.10	0.44 ± 0.15	0.02 ± 0.16	0.43 ± 0.13
**UBLW**	0.50 ± 0.19	0.73 ± 0.07	0.63 ± 0.14	−0.03 ± 0.26	0.18 ± 0.19	0.38 ± 0.04	−0.36 ± 0.18	−0.19 ± 0.11	−0.27 ± 0.13	0.14 ± 0.20	−0.22 ± 0.17
**UBW**	0.12 ± 0.23	0.92 ± 0.03	0.37 ± 0.19	−0.19 ± 0.26	0.13 ± 0.19	0.22 ± 0.12	−0.30 ± 0.21	−0.21 ± 0.19	−0.12 ± 0.24	−0.29 ± 0.20	−0.20 ± 0.19
**UHW**	−0.10 ± 0.18	−0.44 ± 0.13	0.15 ± 0.18	0.21 ± 0.21	−0.44 ± 0.14	0.46 ± 0.05	0.38 ± 0.16	0.55 ± 0.13	0.59 ± 0.28	−0.12 ± 0.16	0.49 ± 0.13
**ULW**	−0.02 ± 0.17	0.71 ± 0.08	0.42 ± 0.14	0.27 ± 0.22	0.06 ± 0.14	0.41 ± 0.10	0.36 ± 0.15	0.62 ± 0.10	0.65 ± 0.15	−0.03 ± 0.15	0.53 ± 0.12
**USW**	0.19 ± 0.20	0.77 ± 0.08 **	0.31 ± 0.17	−0.05 ± 0.22	−0.27 ± 0.16	0.42 ± 0.04	−0.06 ± 0.18	0.11 ± 0.16	0.28 ± 0.20	−0.02 ± 0.17	−0.04 ± 0.16

Results marked with ** had HCW removed from the model in order to achieve convergence. N/A represents when convergence was unable to be achieved. ^1^ BLFT: belly flop test; BL: belly length; BLW: boneless loin weight; BRW: back ribs weight; BW: belly width; BWR: belly width rear; SRW: side ribs weight; SW: sirloin weight; TW: tenderloin weight; TBLW: trimmed belly weight; TBW: trimmed Boston butt weight; THW: trimmed ham weight; TPW: trimmed picnic shoulder weight; UBLW: untrimmed belly weight; UBW: untrimmed Boston butt weight; UHW: untrimmed ham weight; USW: untrimmed shoulder weight; ULW: untrimmed loin weight; ADG: average daily gain; HCW: hot carcass weight; LW: live weight; DP: dressing percentage; GBF: backfat depth; LA: loin area; LC: loin circumference; LL: loin length; RMD: ruler muscle depth.

**Table 11:** Genetic correlations among growth and conventionally measured carcass traits.

Trait ^1^	HCW	LW	DP	GBF	GI	GLD	LA	LC	LL	RMD
**ADG**	0.93 ± 0.04	0.97 ± 0.04	0.44 ± 0.19	−0.14 ± 0.16	0.74 ± 0.78	−0.24 ± 0.16	−0.08 ± 0.15	0.42 ± 0.28	−0.12 ± 0.17	−0.09 ± 0.15
**HCW**		0.90 ± 0.04	0.51 ± 0.17	0.12 ± 0.14	0.79 ± 0.61	−0.36 ± 0.14	−0.66 ± 0.08	−0.63 ± 0.08 **	−0.23 ± 0.14	−0.16 ± 0.14
**LW**			0.17 ± 0.22	−0.02 ± 0.16	0.36 ± 0.67 *	−0.40 ± 0.16	0.00 ± 0.16	0.48 ± 0.33	0.24 ± 0.16	0.00 ± 0.16
**DP**				0.17 ± 0.22	−0.20 ± 0.59	−0.20 ± 0.59	−0.03 ± 0.19	0.03 ± 0.05	−0.30 ± 0.20	0.28 ± 0.25
**GBF**					0.04 ± 0.19	−0.32 ± 0.13	−0.49 ± 0.11	−0.60 ± 0.24	−0.61 ± 0.12	−0.23 ± 0.13
**GI**						−0.23 ± 0.09	0.14 ± 0.10	0.62 ± 0.01	0.10 ± 0.05	−0.04 ± 0.11
**GLD**							0.90 ± 0.06	0.83 ± 0.27	−0.20 ± 0.16	0.91 ± 0.06
**LA**								0.99 ± 0.01	−0.03 ± 0.13	0.86 ± 0.06
**LC**									−0.20 ± 0.28	0.95 ± 0.31
**LL**										−0.23 ± 0.14

Results marked with ** had HCW removed from the model in order to achieve convergence. ^1^ ADG: average daily gain; HCW: hot carcass weight; LW: live weight; DP: dressing percentage; GBF: backfat depth; LA: loin area; LC: loin circumference; LL: loin length; RMD: ruler muscle depth.

**Table 12:** Genetic correlations among pork quality traits.

Trait ^1^	DL2	DLP	LJPC	L*	a*	b*	LNC	LNM	LPHA
**DL1**	N/A	0.21 ± 0.36	−0.03 ± 0.18	0.03 ± 0.17	−0.13 ± 0.18	−0.09 ± 0.16	0.13 ± 0.24	−0.35 ± 0.13	−0.13 ± 0.16
**DL2**		0.18 ± 0.29	−0.01 ± 0.19	0.02 ± 0.18	−0.14 ± 0.18	−0.10 ± 0.17	0.15 ± 0.24	−0.34 ± 0.18	−0.12 ± 0.17
**DLP**			−0.74 ± 0.24	0.70 ± 0.21	0.24 ± 0.24	0.61 ± 0.21	−0.70 ± 0.13	−0.30 ± 0.19	−0.76 ± 0.25
**LJPC**				−0.79 ± 0.02	0.19 ± 0.04	−0.34 ± 0.11	0.96 ± 0.00	0.07 ± 0.13	0.67 ± 0.08
L*					0.47 ± 0.13	0.82 ± 0.06	−0.84 ± 0.13	0.34 ± 0.13	−0.48 ± 0.11
a*						0.86 ± 0.05	0.20 ± 0.24	0.54 ± 0.12	−0.38 ± 0.13
b*							−0.45 ± 0.18	0.45 ± 0.12	−0.59 ± 0.10
**LNC**								−0.07 ± 0.16	0.56 ± 0.08
**LNM**									0.17 ± 0.05

N/A represents when convergence was unable to be achieved. ^1^ DL1: 25 cm chop initial weight; DL2: 25 cm chop post weight; DLP: drip loss percentage; LJPC: Japanese loin color scale; L*: Minolta L*; a*: Minolta a*; b*: Minolta b*; LNC: NPPC loin color scale; LNM: NPPC loin marbling scale; LPHA: loin pH.

**Table 13:** Genetic correlations among novel traits.

**Trait ^1^**	**BL**	**BLW**	**BRW**	**BW**	**BWR**	**SRW**	**SW**		**TBLW**
**BLFT**	−0.66 ± 0.64	−0.21 ± 0.01	−0.36 ± 0.07	−0.53 ± 0.66	0.21 ± 0.64	−0.58 ± 0.15	0.04 ± 0.46		0.37 ± 0.30
**BL**		0.36 ± 0.31	0.33 ± 0.41	−0.12 ± 0.52	0.08 ± 0.06	0.81 ± 0.53	−0.66 ± 0.51		−0.40 ± 0.40
**BLW**			0.20 ± 0.07	0.14 ± 0.54	−0.48 ± 0.33	0.21 ± 0.11	0.52 ± 0.07		−0.01 ± 0.17
**BRW**				−0.37 ± 0.06	0.31 ± 0.06	0.71 ± 0.14	−0.30 ± 0.33		−0.31 ± 0.24
**BW**					0.87 ± 0.83	0.25 ± 0.14	−0.12 ± 0.15		0.19 ± 0.37
**BWR**						0.41 ± 0.09	−0.45 ± 0.05		0.48 ± 0.04
**SRW**							−0.27 ± 0.09		−0.06 ± 0.21
**SW**									−0.19 ± 0.31
	**TBW**	**THW**	**TPW**	**TW**	**UBLW**	**UBW**	**UHW**	**ULW**	**USW**
**BLFT**	−0.47 ± 0.01	−0.87 ± 0.06	−0.58 ± 0.31	−0.25 ± 0.06	0.09 ± 0.38	−0.16 ± 0.37	−0.49 ± 0.14	0.29 ± 0.23	−0.45 ± 0.31
**BL**	−0.23 ± 0.35	0.28 ± 0.33	−0.52 ± 0.71	−0.00 ± 0.49	0.02 ± 0.39	−0.78 ± 0.66	−0.03 ± 0.33	0.05 ± 0.33	−0.65 ± 0.67
**BLW**	−0.08 ± 0.14	0.46 ± 0.10	0.09 ± 0.17	0.30 ± 0.05	0.09 ± 0.17	−0.47 ± 0.17	0.29 ± 0.13	0.72 ± 0.08	−0.21 ± 0.15
**BRW**	0.25 ± 0.07	0.44 ± 0.16	0.32 ± 0.25	0.40 ± 0.17	0.06 ± 0.24	0.04 ± 0.28	0.13 ± 0.20	0.02 ± 0.20	0.31 ± 0.21
**BW**	0.00 ± 0.01	0.30 ± 0.45	0.22 ± 0.08	0.13 ± 0.21	0.30 ± 0.40	−0.24 ± 0.36	0.37 ± 0.18	0.08 ± 0.26	0.01 ± 0.14
**BWR**	0.34 ± 0.45	−0.20 ± 0.39	0.33 ± 0.06	−0.42 ± 0.05	0.70 ± 0.03	0.43 ± 0.09	−0.06 ± 0.06	−0.24 ± 0.10	0.67 ± 0.03
**SRW**	0.24 ± 0.11	0.21 ± 0.14	−0.03 ± 0.23	0.03 ± 0.06	0.41 ± 0.18	−0.11 ± 0.13	−0.06 ± 0.17	0.03 ± 0.16	0.13 ± 0.17
**SW**	0.26 ± 0.19	0.47 ± 0.26	0.02 ± 0.37	0.62 ± 0.04	−0.28 ± 0.31	0.10 ± 0.19	0.42 ± 0.30	0.29 ± 0.20	0.06 ± 0.27
**TBLW**	−0.60 ± 0.16	−0.33 ± 0.17	−0.77 ± 0.30	−0.42 ± 0.17	0.87 ± 0.04	−0.55 ± 0.25	−0.15 ± 0.21	0.30 ± 0.19	−0.72 ± 0.18
**TBW**		0.18 ± 0.14	0.40 ± 0.22	0.23 ± 0.07	−0.43 ± 0.18	0.80 ± 0.05	−0.19 ± 0.16	−0.38 ± 0.16	0.75 ± 0.10
**THW**			0.37 ± 0.16	0.57 ± 0.11	−0.24 ± 0.15	−0.16 ± 0.17	0.88 ± 0.03	0.19 ± 0.14	0.16 ± 0.16
**TPW**				0.21 ± 0.21	−0.71 ± 0.37	0.23 ± 0.23	0.38 ± 0.24	−0.10 ± 0.23	0.77 ± 0.08
**TW**					−0.41 ± 0.18	0.01 ± 0.21	0.34 ± 0.16	0.31 ± 0.07	0.20 ± 0.17
**UBLW**						−0.51 ± 0.26	−0.17 ± 0.21	0.24 ± 0.19	−0.61 ± 0.20
**UBW**							−0.28 ± 0.18	−0.36 ± 0.23	0.82 ± 0.07
**UHW**								0.20 ± 0.16	−0.03 ± 0.19
**ULW**									−0.27 ± 0.18

^1^ BLFT: belly flop test; BL: belly length; BLW: boneless loin weight; BRW: back ribs weight; BW: belly width; BWR: belly width rear; SRW: side ribs weight; SW: sirloin weight; TW: tenderloin weight; TBLW: trimmed belly weight; TBW: trimmed Boston butt weight; THW: trimmed ham weight; TPW: trimmed picnic shoulder weight; UBLW: untrimmed belly weight; UBW: untrimmed Boston butt weight; UHW: untrimmed ham weight; USW: untrimmed shoulder weight; ULW: untrimmed loin weight.

## Supplementary Materials

The following is available online at https://www.mdpi.com/2076-2615/10/5/779/s1. Table S1: Heritabilities and phenotypic and genetic correlations for all traits.
